# Delirium from the gliocentric perspective

**DOI:** 10.3389/fncel.2015.00171

**Published:** 2015-05-11

**Authors:** Adonis Sfera, Carolina Osorio, Amy I. Price, Roberto Gradini, Michael Cummings

**Affiliations:** ^1^Psychiatry, Patton State HospitalCA, USA; ^2^Psychiatry, Loma Linda UniversityCA, USA; ^3^Evidence Based Health Care, University of OxfordOxford, UK; ^4^Pathology, Sapienza UniversityRome, Italy

**Keywords:** immunity, acethylcholine, volume transmission, AQP-4, extracellular space

## Abstract

Delirium is an acute state marked by disturbances in cognition, attention, memory, perception, and sleep-wake cycle which is common in elderly. Others have shown an association between delirium and increased mortality, length of hospitalization, cost, and discharge to extended stay facilities. Until recently it was not known that after an episode of delirium in elderly, there is a 63% probability of developing dementia at 48 months compared to 8% in patients without delirium. Currently there are no preventive therapies for delirium, thus elucidation of cellular and molecular underpinnings of this condition may lead to the development of early interventions and thus prevent permanent cognitive damage. In this article we make the case for the role of glia in the pathophysiology of delirium and describe an astrocyte-dependent central and peripheral cholinergic anti-inflammatory shield which may be disabled by astrocytic pathology, leading to neuroinflammation and delirium. We also touch on the role of glia in information processing and neuroimaging.

## Is Delirium an Astropathy?

The search for cellular and molecular underpinnings of delirium became important as it was demonstrated that after an episode of delirium in elderly, there is a 63% probability of developing dementia at 48 months compared to 8% in patients without delirium^1^ (Pandharipande et al., 2013). Furthermore, an association was shown between delirium and increased mortality, length of hospitalization, cost, and discharge to extended stay facilities (Witlox et al., 2010).

Examined exclusively from the neuronal standpoint, delirium is often described as a neurobehavioral syndrome. In this article we take a gliocentric approach and look at the role of astrocytes in the pathophysiology of delirium. Observed from this angle, delirium is as much a neuro as a gliobehavioral disorder marked by a global cholinergic deficit and a malignant inflammation. We believe that both hypo-cholinergia and inflammation are symptoms of astrocytic failure. Furthermore, aside from reconciling neuroinflammation and cholinergic deficit in the pathogenesis of delirium, adopting a glial perspective explains the astrocytic origin of most delirium markers.

**Figure 1:**
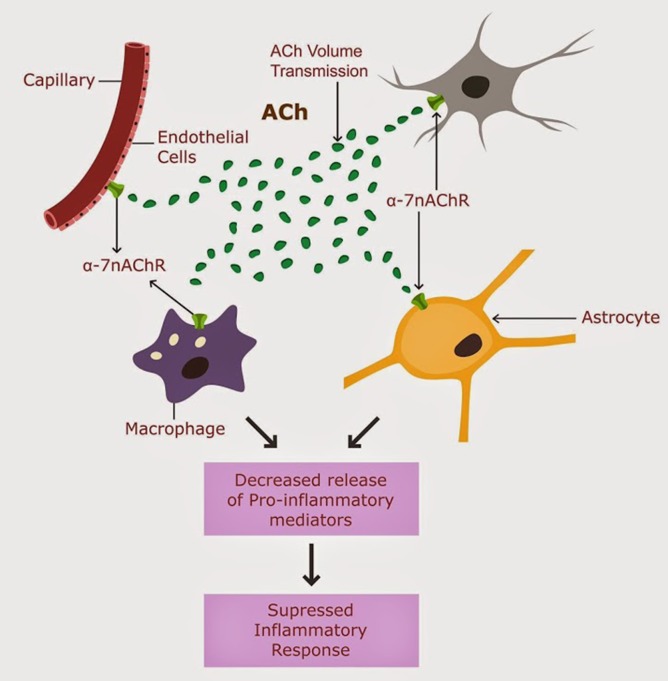
**CAIS is operational in the CNS, and involves ACh action of alpha 7 nAChRs expressed on astrocytes, neurons, microglia and brain macrophages (Tyagi et al., 2010)**.

Acethylcholine (ACh) signaling was suggested to engender a potent cholinergic anti-inflammatory shield (CAIS), protecting against peripheral and central inflammation (Tracey, 2009). CAIS operates in the extracellular space (ECS) both peripherally and centrally by blocking the release of pro-inflammatory mediators (Shaked et al., 2009; Han et al., 2014). CAIS consists of extracellular ACh biosynthesis, its diffusion through the interstitium and action on alpha-7 nicotinic ACH receptors (nAChR) expressed on the peripheral and central immune cells (macrophages, natural killer cells, lymphocytes, astrocytes and microglia), as well as on neuronal and endothelial cells in the CNS (Perry and Teeling, 2013; Figure 1).

The brain is not protected from peripheral pro-inflammatory mediators, as it was believed in the past. In fact, recent studies demonstrate that peripheral cytokines cross routinely the intact blood brain barrier and interact with brain cells. Despite their entry into the CNS, the peripheral pro-inflammatory cytokines are inactivated by CAIS, thus preventing the seeding of inflammation into the brain.

We propose that CAIS is engendered by the astrocytes by secretion of choline acetyltransferase (ChAT), the ACh synthesizing enzyme, into the ECS and by regulation of the interstitial fluid (ISF) volume.

Astrocytes maintain brain water homeostasis by regulating the ISF volume. They accomplish this as their membranes are more permeable to water than those of other brain cells (8). This unique property of astrocytes is based on aquaporine-4 (AQP-4) water channels expressed in large numbers on their end feet processes. In fact, astrocytes express the largest number of AQP-4 receptors in the entire CNS (Nagelhus and Ottersen, 2013). This unique property of their membranes confers astrocytes a sponge-like ability to absorb and release water as needed, thus regulating ISF volume (Gundersen, 2013). With the same token, this property renders astrocytes vulnerable to cytotoxic edema with resultant ISF hypovolemia. As a result of swelling, astrocytes become insufficient, thus unable to secrete ChAT into the ECS which disables CAIS, unleashing neuroinflammation. Moreover, ISF hypovolemia disables beta-amyloid clearance, cluttering the ECS, and disabling CAIS further. Indeed, astrocyte swelling and accumulation of molecular waste in the interstitium was described in several neuro-psychiatric conditions including: sepsis-induced delirium (SID), stroke, traumatic brain injury, brain tumors and metastases, meningitis, brain abscesses, water intoxication, altitude sickness, malignant hypertension, hypoglycemia, and metabolic encephalopathies (Thrane et al., 2014).

We hypothesize that delirium is the clinical manifestation of astrocytic failure caused by astrocytic edema. Edema is the result of AQP-4 water channels’ overexpression on astrocytic membranes. We hypothesize further that failed astrocytes are unable to secrete ChAT into the ECS, disabling CAIS, while the resultant ISF hypovolemia disables beta-amyloid clearance, further impairing CAIS. In the absence CAIS protection, uncontrolled neuroinflammation flares up possibly resulting in delirium. To illustrate this hypothesis, we envision astrocytes as the firefighters of the brain. They keep the fire (neuroinflammation) under control, by maintaining enough water reserves into the interstitial space. In the absence of this water, fire flares up, resulting in uncontrolled neuroinflammation and delirium.

## The Brain-Immune Interface

The fact that the brain is an immunologically privileged organ is a myth. Recent studies demonstrate a continuous two-way communication between the CNS and the peripheral immune system (Perry and Teeling, 2013). For example, it was demonstrated that a cardiac arrest can induce alterations in the central cholinergic signaling, including reduction of ChAT and vesicular ACh transporter mRNA (Norman et al., 2011; Zhao et al., 2013). Conversely, global cerebral ischemia often induces peripheral inflammation (Zhan et al., 2010). This inflammatory response can be pharmacologically reversed by the administration of selective alpha 7 nAChR agonists such as GTS-21, suggesting a role for selective nicotinic agonists in delirium (de Jonge and Ulloa, 2007; Kox et al., 2011).

Alpha 7 nAChRs agonistic ligands are currently being investigated in the treatment of sepsis, inflammation, dementia and schizophrenia (Han et al., 2014). In addition, CAIS was shown to prevent obesity-induced inflammation and insulin-resistance (Lakhan and Kirchgessne, 2011). It is well known that muscarinic M3 receptors in the pancreas are involved in the metabolic syndrome (Duttaroy et al., 2004). This beneficial effect of ACh can be reversed by the administration of alpha 7 nAChR antagonists (Kox et al., 2011). Furthermore, the action of ACh on selective muscarinic receptors was demonstrated to modulate cellular proliferation and is therefore of interest in various cancers (Spindel, 2012; Ferretti et al., 2013). Interestingly, an aberrant neuronal cell cycle entry was suggested in the pathogenesis of Alzheimer’s disease (AD; Stieler et al., 2001).

## Biological Markers: Not too Specific for Delirium, but Specific for Astrocytes

Most biological markers of interest in delirium can be traced to astrocytes. It is well established that Matrix Metaloprotease-9 (MMP-9; Yin et al., 2006), S 100B protein (Tateishi et al., 2006) and glial fibrillary acidic protein (GFAP; Brownell et al., 1991) are secreted by astrocytes. Procalcitonin (McGrane et al., 2011), a newly identified marker of delirium caused by sepsis is expressed on neurons, astrocytes, microglia and oligodendrocytes (Ojeda et al., 2006).

A recent CSF proteomic analysis identified four categories of markers in delirium. Interestingly, they can be traced to the astrocytes as well (Poljak et al., 2014). They include: acute phase proteins, granins, serine protease inhibitors, and apolipoproteins (references in parenthesis demonstrate glial origin).

**Acute phase proteins**: lipocalins (Jang et al., 2013) (includes alpha-1-acidic glycoprotein), alpha-2 microglobulin (Bauer et al., 1988), fibrinogen (Pichler et al., 1999; Hsiao et al., 2013), alpha-1 antitrypsin (Gollin et al., 1992), alpha-1 antichymotrypsin (Gollin et al., 1992), transferrin (Qian et al., 1999), complement component 3 (Maranto et al., 2008), and haptoglobin (Lee et al., 2002).

Interestingly, alpha 1-acidic glycoprotein was shown to decrease brain cellular edema after experimental stroke, possibly by down-regulating AQP-4 receptors (Pichler et al., 1999).

**Granins**: secretogranin 3 (Paco et al., 2010).

**Serine protease inhibitors**: occur both in neurons and glial cells (Buisson et al., 1998).

**Apolipoproteins**: Apo A1 (Ito et al., 2002), ApoJ (DeMattos et al., 2001), ApoE (DeMattos et al., 2001).

These markers point to glial pathology in delirium and are in line with our hypothesis on astrocytic failure.

## Astrocytes and ChAT Secretion

At the cellular level, ACh biosynthesis occurs in two distinct brain compartments: the neuronal bodies of brain cholinergic tracts and the ECS of CNS (Vijayaraghavan et al., 2013). Regardless of their origin, ACh manufacture depends on the availability of its synthesizing enzyme ChAT. Secreted by astrocytes into the ECS, this enzyme ensures a constant presence of ACh in the interstitium (Vijayaraghavan et al., 2013). Figure 1. This ambient ACh is crucial for proper CAIS functioning. Previously it was assumed that ACh could not participate in long distance intercellular signaling because of the presence of its hydrolyzing enzymes, acetylcholinesterase (AChE) and butyrylcholinesterase (BuChE) in the ECS. Recent studies reveal that ACh can “survive” in the ECS and participate in long distance signaling despite physiological levels of AChE and BuChE because it can be synthesized *ad hoc* with astrocyte-secreted ChAT (Vijayaraghavan et al., 2013).

Diminished ACh signaling in the ECS disables CAIS and this event is marked by an immediate compensatory up-regulation of alpha 7 nAChRs, the first sign of inflammation. We believe that alpha 7 nAChRs up-regulation is the first measurable marker of astrocytic failure. Indeed, a recent study demonstrates that increased astrocytic expression of alpha 7 nAChRs is positively correlated with the extent of neuropathological alterations in AD (Yu et al., 2012). Measuring ECS level of ChAT may provide a biological marker of pending inflammation as it expresses alpha 7 nAChRs up-regulation.

Astrocytes contribute to beta-amyloid clearance either directly, by phagocytosis, or indirectly by the glymphatic system (Nagele et al., 2003; Lasagna-Reeves and Kayed, 2011; Sokolowski and Mandell, 2011; Iliff et al., 2012). We believe that swollen, failed astrocytes may be inefficient in disposing of beta amyloid and other molecular waste, leading to their accumulation and neuroinflammation. Indeed, studies in AD reveal that excess beta-amyloid in the ECS alters cholinergic signaling by activating BuChE, decreasing secretion of astrocytic ChAT and up-regulating alpha 7 nACh receptors on astrocytic membranes (Vijayaraghavan et al., 2013; Malmsten et al., 2014).

Beta amyloid clearance via the glymphatic system is highly dependent on the intra-parenchymal water exchange between the cerebrospinal fluid (CSF) and the ISF, occurring via AQP-4 channels in astrocytic end-feet (Yang et al., 2013) Figure 2.

**Figure 2:**
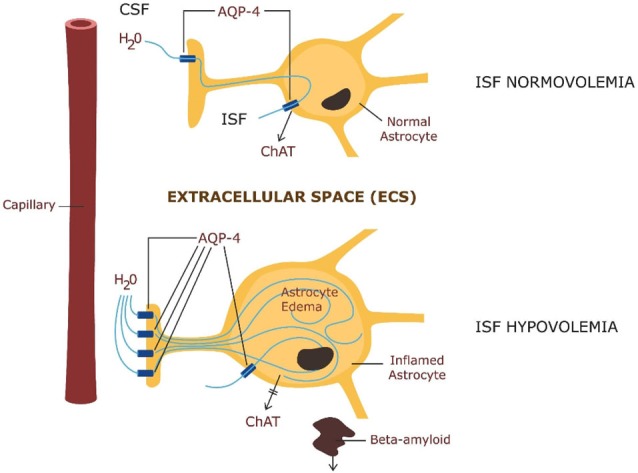
**ABOVE: Astrocytic physiology—intra-parenchymal CSF exchange with the interstitial fluid (ISF) via AQP-4 channels and ChAT secretion**. BELOW: Pathology—astrocytic edema: up-regulation of AQP-4 in astrocytic end-feet with impairment of ChAT secretion and beta amyloid clearance.

Accumulation of beta-amyloid and low ACh were documented in delirium (Hshieh et al., 2008) and led to the emission of the cholinergic hypothesis in the pathogenesis of this condition. However, since cholinesterase inhibitors showed only mild or no efficacy in delirium, this hypothesis seemed to have been invalidated (van Eijk et al., 2010; Brinker et al., 2014).

Astrocytic failure hypothesis offers an alternative explanation that does not necessarily invalidate the cholinergic hypothesis. On the contrary, since they do not restore astrocytic secretion of ChAT, cholinesterase inhibitors are not expected to beneficial in delirium. In the absence of ACh biosynthesis in the ECS, inhibiting its hydrolyzing enzymes would not resurrect CAIS function. On the other hand, co-administration of cholinesterase inhibitors with drugs capable of restoring the extracellular water volume (such as agonists of alpha 7 nAChRs, direct AQP-4 blockers or indirect ones, such as erytropoietin) may be salutary.

## Is there a Water Connectome?

Being more permeable to water, astrocytes serve as brain fluid reservoirs where water can be stored and also retrieved as needed (Gundersen, 2013). The high AQP-4 expression on their membranes enables astrocytes to move water in and out of the neuropile. Studies done three decades ago show that water homeostasis is dependent on the neuropile activity (Dudek and Rogawski, 2005). For example, during the day (when information processing is usually more intense), water is shifted into the astrocytic compartment, while during the night (when there is less information processing), water is shifted back into the interstitium (Xie et al., 2013). This water movement was hypothesized to help beta amyloid clearance during sleep (Brinker et al., 2014).

Water can cross cellular membranes slowly by diffusion or co-transport with other substances, but the quickest modality of water transport occurs via AQP-4 receptors which abound on astrocytic membranes (Gundersen, 2013). The high permeability of astrocytic membranes and the sponge-like properties of these cells render neuroimaging possible. Diffusion tensor imaging (DTI) visualizes water diffusion across membranes which can be either unrestricted (isotropy) or restricted by the myelin sheath (anisotropy). Since water does not diffuse through the myelin, but follows it, this technique is used for tracing white matter tracts (Lazar et al., 2003; Lazar, 2010).

We suggest that DTI could be utilized for assessing glial integrity by adapting its algorithm to the areas of highest isotropy. Since astrocytes are the most water permeable cells in the brain, the highest CNS isotropy must coincide anatomically with astrocytes.

Recent data point to the fact that DTI signal changes, attributed earlier to white matter tracts and axonal myelination, may in fact mirror astrocytic expression of AQP-4 receptors (Meng et al., 2004; Tourdias et al., 2009), astrocytic edema (Harsan et al., 2007; Fukuda and Badaut, 2012), ISF volume (Fukuda and Badaut, 2012), or glial scars (Budde et al., 2011).

In its journey through the brain fluid compartments, water carries along signaling molecules that comprise an extra-cellular communication platform described as volume transmission (VT; Agnati et al., 1995). Does this signaling platform fathom a liquid connectome? Furthermore, since it mirrors the water flow along white matter tracts, is the connectome in general a liquid connectome? It was proposed that point-to-point synaptic transmission engenders a quick and precise mode of information processing, such as the one necessary for playing tennis or piano, while the diffuse and widespread communication platform by water signaling may engender more pervasive functions such as awareness, attention, mood, circadian rhythm or cognition (Gualtieri, 2002; Taber and Hurley, 2014; Vizi et al., 2010). As these functions are known to “wax and wane” in delirium, can they be part of an astrocyte-centered information processing? Recent studies imply that astrocytic networks are crucial for information integration and cholinergic plasticity (Pereira and Furlan, 2010; Takata et al., 2011). A recent study demonstrated that ACh signaling is highly correlated with gamma oscillations on the EEG, which are believed to facilitate the somatosensory cortex plasticity (Rodriguez et al., 2004). This may suggest an astrocytic-based cognition, possibly complementary to the rapid, point-to-point information processing established by neurons.

## Conclusions

Memory characterizes both, cellular immunity and human cognition. Both systems are adapted to interact with the surrounding world and to protect from it. Both are capable of distinguishing “self” from the environment (“non-self; Lampron et al., 2013). Since nature rarely wastes ideas, immunologic memory may be a distant phylogenetic ancestor of human cognition. Controlled inflammation and cellular proliferation may benefit hippocampal neuroplasticity and long term potentiation. Out of control, inflammation, on the other hand, may lead to sepsis, while aberrant cellular proliferation to cancer. With the same token, activation of cellular proliferation in non-dividing cells, like neurons, may lead to aberrant cell cycle entry, resulting in apoptosis. This may be the case in AD.

Both astrocytes and microglia are part of the innate immune system which responds to the early stages of infection by triggering non-specific, high collateral damage, inflammatory responses, which are later expected to die down, as the high precision, specific, adaptive immune system is activated. Failure of the innate system to shut down may result in “cytokine storms” encountered in sepsis and sepsis-induced delirium (SID). On the other hand, intact astrocytes with proper CAIS calm down the innate immune system, containing inflammatory responses. Failed astrocytes unleash exaggerated inflammation which may lead to delirium. Engel and Romano (1959) described delirium as “brain failure”; we think of it as a primary astrocytic failure with secondary neuronal involvement. Since astrocytic suffering occurs prior to neuronal damage, identifying it early may offer a window of opportunity for preventive interventions that could salvage the cognitive domain before the occurrence of neuronal apoptosis.

## Conflict of Interest Statement

The authors declare that the research was conducted in the absence of any commercial or financial relationships that could be construed as a potential conflict of interest.

## Footnotes

^1^American Delirium Society. https://www.americandeliriumsociety.org/about-delirium/healthcare-professionals, available on line.
